# Keratin 13 is a more specific marker of conjunctival epithelium than keratin 19

**Published:** 2011-06-18

**Authors:** Arturo Ramirez-Miranda, Martin N. Nakatsu, Siamak Zarei-Ghanavati, Christine V. Nguyen, Sophie X. Deng

**Affiliations:** 1 Cornea and Uveitis Division, Jules Stein Eye Institute, University of California, Los Angeles, CA; 2 Georgetown University, School of Medicine, Washington, DC

## Abstract

**Purpose:**

To evaluate the expression patterns of cytokeratin (K) 12, 13, and 19 in normal epithelium of the human ocular surface to determine whether K13 could be used as a marker for conjunctival epithelium.

**Methods:**

Total RNA was isolated from the human conjunctiva and central cornea. Those transcripts that had threefolds or higher expression levels in the conjunctiva than the cornea were identified using microarray technique. Expression levels of three known signature genes and of two conjunctival genes, *K13* and *K19* were confirmed by using quantitative real-time PCR (qRT–PCR). Protein expression of K12, K13, and K19 was confirmed by immunostaining with specific antibodies on histologic sections of human sclerocornea that contained the conjunctiva, limbus, and cornea and on impression cytology (IC) specimens of the cornea and conjunctiva from normal donors. Double staining of K13/K12 and K19/K12 on histologic sections and IC specimens was performed.

**Results:**

There were 337 transcripts that were preferentially expressed in the conjunctiva. *K13* and *K19* were among the top twenty transcripts in the conjunctiva and this preferential expression pattern of *K13* and *K19* was confirmed by qRT–PCR. Immunohistochemical studies showed that K13 was expressed at the posterior limbal epithelium and conjunctival epithelium but was totally absent in the cornea. K12 was expressed in the corneal and anterior limbal epithelia except for the basal layer and was absent from the conjunctiva. In contrast, K19 was detected in the corneal, limbal and conjunctival epithelia. Immunostaining of the IC specimens showed K12^+^ epithelial cells in the corneal region, K13^+^ cells in the conjunctival area, and K19^+^ cells in the corneal and conjunctival specimens. Expression of K13 and K12 on the ocular surface was mutually exclusive on both the histologic and IC samples using double immunostaining.

**Conclusions:**

K13 is more specific to the conjunctival epithelial cells than K19 and potentially could be used as a marker to identify conjunctival epithelial cells in limbal stem cell deficiency.

## Introduction

The ocular surface is lined by the corneal and conjunctival epithelia. Despite having different phenotypes and histogeneses, both epithelia form a continuous layer and function as barriers to protect the ocular surface from injury, infection, and desiccation. The human corneal epithelial stem cells (limbal stem cells [LSCs]) are presumed to locate in the limbus, ie, the transitional zone between the cornea and the conjunctiva, and they maintain the homeostasis of corneal epithelial cells [1,2]. When LSCs are damaged or deficient, the conjunctival epithelium invades the corneal surface, and this invasion leads to corneal opacity and neovascularization [3]. This conjunctivalization process on the corneal surface severely impairs vision and causes blindness at the end stage [4].

Diagnosis of LSC deficiency (LSCD) is made by clinical examination and confirmed by impression cytology (IC) [5]. The presence of goblet cells on the cornea indicates the presence of conjunctival epithelium. However, in many ocular disorders in which LSCD is observed, goblet cell deficiency also coexists, and this coexistence leads to a false-negative result. Egbert et al. [6] found that it is difficult to distinguish conjunctival epithelia from corneal epithelia by conventional cytology techniques.

The identification of a marker that is expressed in the conjunctival epithelium but not in the corneal epithelium has been a growing need. Good candidates are cytokeratins, which comprise a family of intermediate filament cytoskeletal proteins in epithelial cells and are divided into the type I (acidic) and type II (basic to neutral) subfamilies. Cytokeratins form filaments responsible for the integrity of the epithelial cell structure, and because of their different patterns of expression, these proteins could be used as differentiation markers [7]. The corneal epithelium, but not the conjunctival epithelium, expresses cytokeratin (K) 12 [8]. Although K19 was proposed initially by Donisi et al. [9] as a specific marker of conjunctival epithelial cells and used by others to diagnose LSCD, other groups found that K19 is not specific to conjunctival epithelial cells because it is expressed in corneal epithelial cells as well [10-12]. Barbaro et al. [13] recently compared K12 and K19 expression in both sclerocorneal tissues and IC specimens and their results confirmed the previous finding that K19 is not specific to conjunctival epithelial cells. A more specific marker of limbal and conjunctival epithelia would be necessary to detect non-corneal epithelial cells on the corneal surface.

To search for conjunctival specific marker(s), we first performed preferential gene profiling in the conjunctiva in direct comparison to that in the cornea using microarray technique. *K13* and *K19* transcripts were among the genes preferentially expressed in the human conjunctiva and their expression levels were confirmed using qRT–PCR. The detailed expression patterns of K13, K19, and K12 on the human ocular surface were then compared by immunohistochemistry and confirmed on impression cytology specimens. K13 was expressed only in the posterior limbal and conjunctival epithelia and completely absent on the cornea.

## Methods

### Human sclerocorneal tissue

Human sclerocorneal tissue of nine healthy donors (age range, 2 to 62 years) was obtained from the Lions Eye Institute for Transplant and Research (Tampa, FL), the Tissue Bank International (Baltimore, MD), and the San Diego Eye Bank (San Diego, CA). The experimental protocol was evaluated and approved by the Institutional Review Boards of the University of California, Los Angeles. Six donor tissues were used in the immunohistochemical study. The death to preservation time was less than 8 h, and the time to tissue processing was less than 4 days. For RNA isolation, the death to preservation time was less than 6 h and the tissues were either snap frozen on dry ice or stored in RNAlater (Ambion Inc., Austin, TX). Three donors were used in the microarray experiment.

### RNA isolation and microarray analysis

The conjunctival and corneal epithelia along with their immediate adjacent stroma were dissected from human sclerocorneal tissues. RNA was isolated as described previously [14]. Briefly, tissues were homogenized in RLT lysis buffer (Qiagen, Valencia, CA) and total RNA from tissues was extracted with the Qiagen RNeasy Mini Kit (Qiagen). The quantity and quality of total RNA were assessed by a NanoDrop 1000 spectrophotometer (NanoDrop, Wilmington, DE) and a 2100 Bioanalyzer (Agilent Technologies, Santa Clara, CA). Only those samples that had an RNA integrity number >9 and exhibited minimal RNA degradation were used for subsequent experiments. Microarray analysis was performed as previously described [14]. Briefly, one transcription amplification was performed. Synthesis for all samples was successful and provided a sufficient yield of cRNA. Affymetrix U133 plus 2.0 human expression arrays (Affymetrix, Santa Clara, CA) was used in accordance with the standard Affymetrix protocol for eukaryotic expression arrays. All microarrays were scanned by using an Affymetrix 3000 one-color microarray scanner. Raw images were examined for surface defects and for proper grid placement. Background intensity, housekeeping gene expression, and a 3′-to-5′ ratio of probe sets for genes of varying lengths were also used to assess the quality. Probe intensity values were generated by using the Affymetrix Gene Chip Operating System. The gene whose expression in the conjunctiva was at least threefold higher than that in the cornea and whose level was ≥145 (1% of the highest expression level in the conjunctiva) was considered to be differentially expressed. We have deposited the raw data at Gene Expression Omnibus (GEO) under accession number GSE29402 and we can confirm all details are Minimum Information About a Microarray Experiment (MIAME) compliant.

### Quantitative RT–PCR

Total RNA was reverse-transcribed by using Superscript II RNase H2 reverse transcriptase (RT; Invitrogen, Carlsbad, CA) according to the manufacturer's recommendations. The relative abundance of transcripts was detected through qRT–PCR by using a Brilliant SYBR Green qRT–PCR Master Mix (Stratagene, La Jolla, CA). The protocol used an Eppendorf realplex2 real-time PCR system (Hamburg, Germany). The primers used for qRT–PCR are listed in Table 1. Cycling conditions were as follows: an initial denaturing step of 10 min at 95 °C and subsequent 40 cycles of amplification in which each cycle consisted of 45 s at 95 °C, 30 s at 55 °C, and 15 s at 72 °C. To generate a dissociation curve after the amplification cycles, each sample was incubated at 95 °C for 15 s and then subjected to a melting curve program (60–95 °C). The fluorescence intensity of each sample was acquired during the execution of the melting curve program and normalized in relation to that of the housekeeping gene, glyceraldehyde-3-phosphate dehydrogenase (*GAPDH*). The average value of triplicates from each transcript was used for comparison.

**Table 1:** qRT–PCR primers.

**Gene**	**Direction**	**Primer sequence**
*K12*	Forward	CCAGGTGAGGTCAGCGTAGAA
* *	Reverse	CCTCCAGGTTGCTGATGAGC
*K13*	Forward	CTGAACAAGGAGGTGTCTACCA
* *	Reverse	ATAGCGGCACTCCGTCTCT
*K15*	Forward	ACCACCACATTTCTGCAAACT
* *	Reverse	AGCTGAGATACTTCGGCTTCC
*K19*	Forward	TGAGTGACATGCGAAGCCAAT
* *	Reverse	ACCTCCCGGTTCAATTCTTCA
*Mucin 5AC*	Forward	CAGCCACGTCCCCTTCAATA
* *	Reverse	ACCGCATTTGGGCATCC
*GAPDH*	Forward	GGCTGAGAACGGGAAGCTTGTCAT
* *	Reverse	CAGCCTTCTCCATGGTGGTGAAGA

Abbreviations: Cytokeratin, K; Glyceraldehyde 3-phosphate dehydrogenase, *GAPDH*.

### Immunohistochemistry

Human sclerocorneal tissues were cut into four quadrants and embedded in Optimal Cutting Temperature Compound (OCT; Tissue-Tek, Torrance, CA) on dry ice. Tissues were cut into 6–8 μm sections with a cryostat and stored at −80 °C. The primary and secondary antibodies used are listed in Table 2. Frozen section slides were warmed in a desiccator at room temperature, fixed with 4% paraformaldehyde for 15 min, washed with phosphate-buffered saline (PBS) three times, and blocked with 5% normal donkey serum (Jackson ImmunoResearch Laboratories, West Grove, PA) in PBS for 30 min. The slides were washed with 1% BSA (BSA)/PBS three times and incubated with the primary antibodies for 60 min at room temperature. The slides were washed with 1% BSA/PBS three times and subsequently incubated with the appropriate secondary antibody. Afterward, the slides were washed with 1% BSA/PBS three times, and the nuclei were labeled with Hoechst 33342 (0.5 μg/ml) for 15 min. The slides were washed with PBS five times and mounted. For double staining, the slides were incubated with the primary antibody for 1 h at room temperature, washed with 1% BSA/PBS three times, and incubated with the appropriate secondary antibody. The slides were then incubated with the second primary antibody for 1 h at room temperature, washed with 1% BSA/PBS three times, incubated with the appropriate secondary antibody for 1 h at room temperature, and washed with 1% BSA/PBS three times. Nuclei were labeled with Hoechst 33342 as above. The slides were washed with PBS five times and mounted. Pictures were taken under a 25× objective lens by using a Zeiss fluorescent microscope (Oberkochen, Germany).

**Table 2:** Primary and secondary antibodies.

**Protein**	**Company**	**Host**	**Species reactivity**	**Clone/ catalog #**
Cytokeratin 12	Santa Cruz Biotechnology, Santa Cruz, CA	goat	human	sc-17101
Cytokeratin 13	Santa Cruz Biotechnology, Santa Cruz, CA	mouse	Human	c-101460
Cytokeratin 19	Dako North America Inc., Carpinteria, CA	mouse	human	RCK108
Cytokeratin 19	Leica Microsystems INC, Bannockburn, Il	mouse	human	b170
Alexa Fluor 488 IgG	Invitrogen, Carlsbad, CA	donkey	goat	A11055
Alexa Fluor 546 IgG	Invitrogen, Carlsbad, CA	donkey	mouse	A10036

### Impression Cytology

A sterile, round, single-packed Biopore 0.45-µm membrane (Millipore Corp., Bedford, MA) was placed on the cornea of the sclerocorneal tissues. Gentle pressure was applied for a few seconds. The membrane was then peeled off. To obtain conjunctival epithelial cells, the membrane was applied to the conjunctiva only in the same manner. The membranes that contained epithelial cells were then fixed with 4% paraformaldehyde for 15 min, washed with PBS three times, and blocked with 5% normal donkey serum (Jackson ImmunoResearch Laboratories) in PBS for 30 min. The membranes were immunostained with anti-K12, anti-K13, and anti-K19 antibodies as described above.

### Data analysis

All Affymetrix data were normalized by using the justRMA algorithm of R software from the Bioconductor group [15], which implements the RMA (robust multiarray average) normalization method [16]. In this normalization step, each array was individually normalized by combining it with a pool of 50 fixed reference arrays in the Microarray Core Facility at the University of California, Los Angeles. Genes whose expression values were at least threefold greater than those in the other tissue type were selected and considered to be differentially expressed. DAVID (the database for annotation, visualization and integrated discovery) was used for functional analysis [17]. Lists of differentially expressed genes were checked by DAVID to find the most over-represented gene groups. The data was obtained from 3 different donors.

### Statistical analysis

To eliminate the variation between experiments in the qRT–PCR, the absolute expression value (highest for either the conjunctiva or cornea) was set at 1 and the ratios of absolute values were calculated between tissues and averaged. A Wilcoxon Signed-rank test was performed on the ratio values and a Student’s *t* test on the quantitation of cell populations. A p value <0.05 was considered statistically significant.

## Results

The RNA quality assessed by using the nano chip revealed a flat baseline with no significant tailing of the rRNA bands (data not shown) and the S18-to-S23 ratios were between 1.6 and 2.1. The RNA isolated from all tissues appeared to have little degradation.

There were 337 transcripts predominantly overexpressed in the conjunctiva. Of the 337 preferentially expressed transcripts in the conjunctiva, 331 encoded proteins with known functions. The top 20 transcripts that were preferentially overexpressed in the conjunctiva are listed in Table 3. Interestingly, both *K19* and *K13* were among the preferential intermediate filaments in the conjunctiva.

**Table 3:** The top 20 preferentially expressed transcripts in the conjunctiva.

**Gene Title**	**Public ID**	**Gene symbol**	**Fold-change**	**Expression level in conjunctiva**	**Expression level in cornea**
ceruloplasmin (ferroxidase)	NM_000096.3	CP	217.38	3669.56	16.88
polymeric immunoglobulin receptor	NM_002644.3	PIGR	176.51	3162.08	17.91
odd-skipped related 2 (Drosophila)	NM_001142462.1	OSR2	144.93	2809.3	19.38
carcinoembryonic antigen-related cell adhesion molecule 7	NM_006890.3	CEACAM7	136.73	4018.48	29.39
S100 calcium binding protein A8	NM_002964.4	S100A8	121.32	14462.8	119.21
S100 calcium binding protein A9	NM_002965.3	S100A9	114.93	13037.13	113.44
aquaporin 5	NM_001651.2	AQP5	92.56	3820.75	41.28
tyrosinase-related protein 1	NM_000550.2	TYRP1	89.69	2980.23	33.23
S100 calcium binding protein P	NM_005980.2	S100P	71.45	6097.61	85.35
phospholipase A2, group IIA (platelets, synovial fluid)	NM_000300.3	PLA2G2A	67.49	3722.1	55.15
complement component 3	NM_000064.2	C3	62.03	4090.82	65.95
carcinoembryonic antigen-related cell adhesion molecule 6 (non-specific cross reacting antigen)	NM_002483.4	CEACAM6	56.78	3162.51	55.7
lipocalin 2	NM_005564.3	LCN2	55.21	6564	118.89
keratin 13	NM_153490.2	KRT13	50.96	12892.51	252.97
secretory leukocyte peptidase inhibitor	NM_003064.2	SLPI	43.63	10857.97	248.89
insulin-like growth factor binding protein 3	NM_001013398.1	IGFBP3	43.4	2665.43	61.42
complement factor D (adipsin)	NM_001928.2	CFD	37.9	10290.04	243.75
arachidonate 5-lipoxygenase	NM_000698.2	ALOX5	36.47	3527.66	93.09
keratin 19	NM_002276.4	KRT19	28.05	2370.14	64.98
retinoic acid receptor responder (tazarotene induced) 1	NM_206963.1	RARRES1	25.04	12176.47	434.09

**Figure 1:**
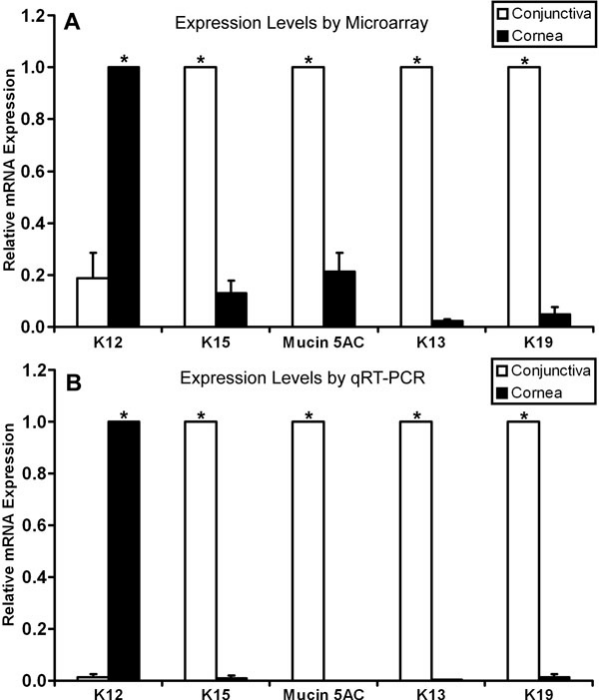
Expression levels of signature genes and of K13 and K19 in the cornea and conjunctiva. Expression levels were obtained by microarray method (**A**) and detected by qRT–PCR (**B**). K12 expression was restricted to the cornea as expected, whereas K15 and Mucin 5AC, both conjunctival markers, were expressed almost exclusively in the conjunctiva. Both *K13* and *K19* transcripts were also preferentially expressed in the conjunctiva. Abbreviations: K, cytokeratin.

To validate our microarray data, we analyzed the expression pattern of several well known signature genes in the cornea and conjunctiva from the global mean intensity values. One of the cornea epithelium markers, keratin 12 [2] was highly expressed in the cornea. Keratin 15, which had been shown to be exclusively expressed at the basal epithelial layer of the limbus and conjunctiva [10], had a higher transcription level in the conjunctiva than cornea (Figure 1A). Mucin 5AC, a conjunctiva marker [18], was also preferentially expressed in the conjunctiva but not in the cornea (Figure 1A). The expression levels of all of these signature genes of each tissue type were highly correlated with their expected expression patterns. In addition to the expected marker expression, both *K13* and *K19* expression were also significantly upregulated in the conjunctiva in comparison to the cornea.

To further verify the microarray method, the five transcripts with differential expression patterns seen in our microarray analysis were independently quantified by qRT–PCR. The expression levels of all five transcripts measured by qRT-PCR correlated well with those obtained using the microarray technique (Figure 1B). *K13* and *K19* were included in this analysis to confirm their expression specificity. The levels of *K13* and *K19* transcripts were significantly higher in the conjunctiva than in the cornea (both p<0.05). *K13* and *K19* mRNA levels were barely detected in the cornea (Figure 1B). This observation suggested that *K13* could be a candidate conjunctival epithelial marker.

**Figure 2:**
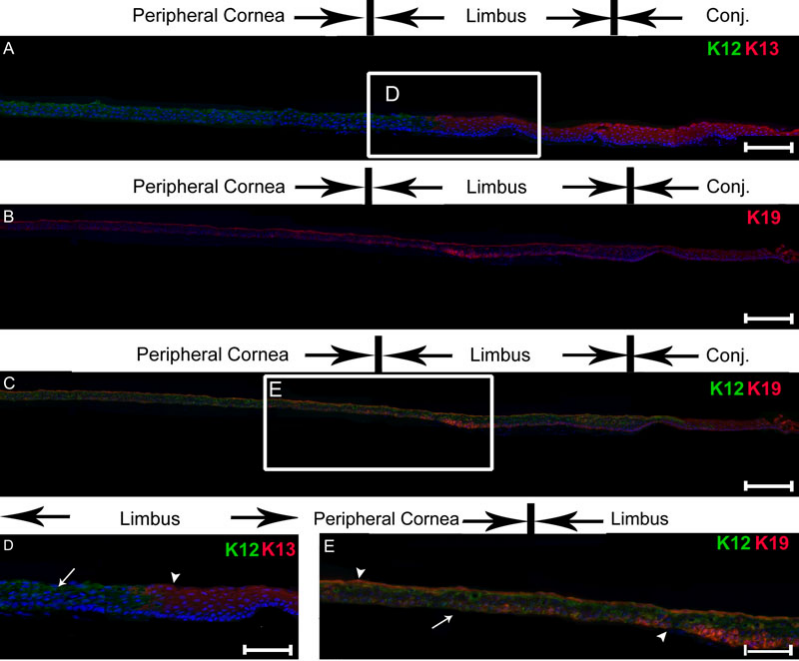
Immunohistochemical analysis of expression patterns of K12, K13, and K19 in normal human histologic sections. Montage images of serial sections of the central cornea to the conjunctiva (**A**-**C**): Double staining of K12 (green) and K13 (red) showed the presence of K13 in the epithelia of the posterior limbus and conjunctiva (**A**). K19 expression was detected in the peripheral cornea, limbus, and conjunctiva (**B**). Double staining of K12 (green) and K19 (red) showed the overlapping expression of both cytokeratins (**C**). **D**: Expression of K12 (arrows) and K13 (arrowheads) were mutually exclusive. **E**: Details of the overlapping expression of K12 and K19. Arrowhead: cells that expressed both K12 and K19. Arrow: K12-expressing cell. Abbreviations: Conj., conjunctiva. Magnification bar in **A**, **B**, **C** represents 200 μm. Magnification bar in **D** and **E** represents 50 μm.

Immunohistochemistry was then used to examine the presence of K13 and K19 in normal human ocular tissues. A montage of images consisting of the conjunctiva, limbus, peripheral cornea and central cornea was constructed to show the detailed expression pattern. As shown in Figure 2A, K13 protein was expressed in the suprabasal limbal epithelium and in all layers of the conjunctival epithelium but was absent in all layers of the corneal epithelium, including the central and peripheral areas. As expected, K12 protein expression was detected in all layers of the corneal epithelium and the suprabasal layers of the limbus, but was not observed in the conjunctival epithelium (Figure 2A). K19 was observed in all layers of the epithelium in the limbus, and the expression extended to the mid-peripheral cornea, although the intensity of the fluorescence toward the center of the cornea decreased (Figure 2B). Only occasionally, the central corneal epithelial cells expressed K19.

**Figure 3:**
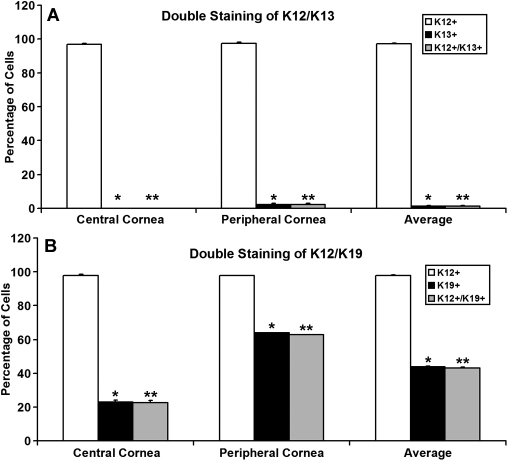
Specificity of K12, K13, and K19 expression in human cornea. **A**: Percentage of K12^+^, K13^+^ and K12^+^/K13^+^ cells in the central (left) and peripheral cornea (middle). The average percentages of the three cell populations are obtained (right). **B**: Percentage of K12^+^, K19^+^ and K12^+^/K19^+^ cells in the central (left) and peripheral cornea (middle). The average percentages of the three cell populations are obtained (right). The asterisk indicates a p<0.05 between K12^+^ and K19^+^ or K13^+^ cells, the double asterisk indicates a p<0.05 between K12^+^ and K12^+^/19^+^ or K12^+^/K13^+^ cells.

To further evaluate the specificity of the expression of K13 and K19, double immunostaining of the sclerocorneal tissue sections was performed. There was a rather abrupt transition between the K12-expressing and K13-expressing epithelial cells in the limbus (Figure 2 A,D). The limbal epithelial cells expressed either K12 or K13. Few cells expressed both K12 and K13; this finding suggested that expression of K12 and K13 is mutually exclusive. In contrast, epithelial cells that expressed both K12 and K19 were located throughout the limbus and the peripheral cornea (Figure 2B,C,E). Quantitation of each population was performed in the central and the peripheral cornea (Figure 3). In the central cornea, nearly all epithelial cells were K12^+^ and 22.6% of them also weakly expressed K19 (Figure 3B). In the peripheral cornea, 97.8% of cells were K12^+^ and 62.7% of them also were K19^+^ (Figure 3B). On average, 97.8% of epithelial cells expressed K12 and 42.9% of them also expressed K19 in the cornea (Figure 3B). In contrast, none of the K12^+^ cells expressed K13 in the central cornea and there was no K13 expression in the peripheral cornea (Figure 3A). The statistical differences between the K12^+^ cells compared to either the K13^+^ or K19^+^ cells were significant for both the peripheral and central cornea.

**Figure 4:**
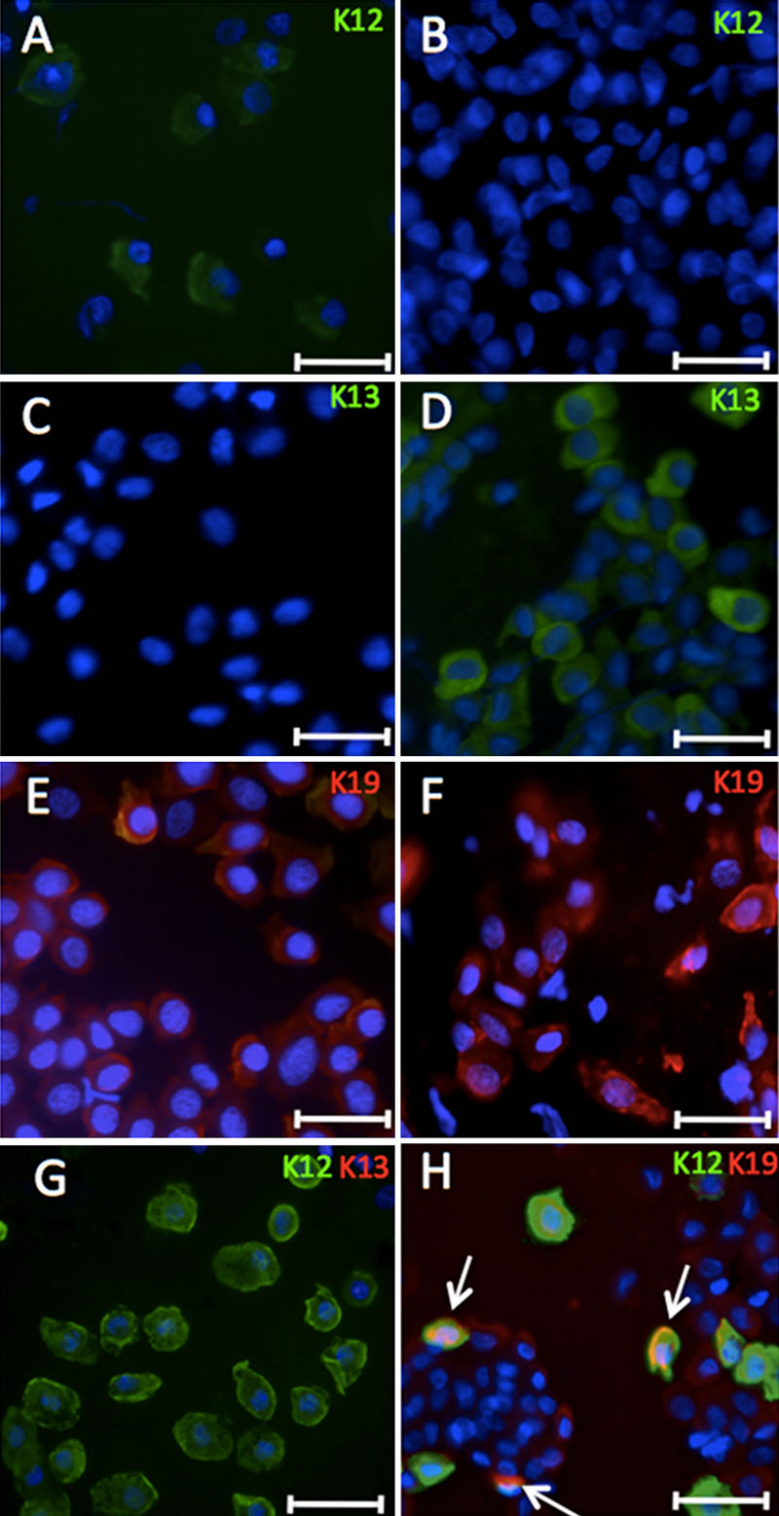
Patterns of K 12, 13, and 19 expression in the impression cytology (IC) specimens taken from normal sclerocorneal tissues. **A**, **C**, **E**, **G**, and **H** were from corneal IC specimens. **B**, **D**, and **F** were conjunctival IC specimens. Expression of K12 was present in the corneal (**A**) but not in the conjunctival epithelium (**B**). K13 expression was not detected in corneal epithelium (**C**) but was highly expressed in the conjunctival (**D**) epithelium. K19 was detected in the both corneal (**E**) and conjunctival (**F**) epithelia. Double staining of K12/K13 (**G**) and K12/K19 (**H**) in corneal IC specimens. Arrows: K12^+^/K19^+^ cells. Magnification bar represents 5 μm.

The specificity of K12, K13, and K19 expression was confirmed on IC specimens of healthy donors. K12 was detected only in IC specimens from the cornea (Figure 4A), but not in the conjunctiva (Figure 4B). K13 was present in IC specimens from the conjunctiva (Figure 4D) but was completely absent in corneal IC specimens (Figure 4C). In contrast, K19 was seen in the conjunctival and corneal IC specimens (Figure 4E,F). Double staining confirmed the K12^+^/K19^+^ cells in the cornea (Figure 4H).

## Discussion

The identification of K13 and K19 in conjunctiva from the microarray analysis and subsequent confirmation through qRT–PCR and immunostaining strongly validates the comparative gene expression analysis from the microarray data. A similar gene expression profile study by Turner et al. [19] using human genome U133A microarrays, containing 22,283 probe sets, compared RNA samples isolated from conjunctival and cornea epithelial sheets. Interestingly, almost all of our preferentially expressed genes in the conjunctiva, including *K13* and *K19*, were present in their conjunctiva exclusive or preferential transcript sets, thus reaffirming the validity of our microarray results despite the slight difference in the ocular tissues used.

This study compares the expression specificity of K13 and K19 at both the mRNA and protein levels on the human ocular surface. Although K19 is expressed at a much lower level in the central cornea, the finding that K19 was detected in 62.7% of the corneal epithelial cells in the peripheral cornea is striking. These observations strongly suggest that K19 is not specific to the conjunctival epithelium. Using qRT–PCR, we detected a low level of *K19* transcripts in the cornea and confirmed the accuracy of our immunostaining results. The qRT–PCR result probably reflects *K19* expression in the peripheral cornea. Our finding is consistent with those reported previously [10,13,20]. The discrepancy in K19 expression may be due to the immunohistochemistry technique employed. The length of fixation, the antibody used, the location of the observation, the quality of specimen, and the sensitivity of immunofluorescence detection might affect the outcome of the immunostaining pattern. Over fixation tends to decrease antibody-antigen binding and hence reduce detection sensitivity. Only fresh tissues with less than 8 h of death to preservation time were used in our experiments. We also employed two different anti-K19 antibodies, and both gave the same immunostaining pattern.

K13, a major acidic keratin, is expressed in the suprabasal layers of non-cornified stratified epithelia and is mucosa-specific [21]. In addition, K13 is present in the suprabasal layers of most stratified squamous epithelia, such as mucosal epithelia and regenerating epidermis [22,23]. Previous studies by us and others showed that K13 is expressed in the conjunctival and limbal epithelia in histological sections [14,20,24] and in cultured tissue [25], but the detailed expression pattern on the central and peripheral cornea, and the limbus has not been studied. This study shows the detailed expression pattern of K13 and K19 in the central and peripheral cornea, and the limbus. The finding that K12 expression and K13 expression are mutually exclusive on the ocular surface makes K13 a potential marker of epithelial cells that are not of corneal phenotype. This is particularly important because the clinical signs in the early stage of LSCD tend to be subtle and not specific. Goblet cells are not always present and could be missed by the standard IC method. The detection of non-corneal epithelial cells, particularly in the peripheral cornea, would be a more sensitive and specific method of diagnosing early LSCD. Furthermore, the successful detection of K13 and K12 by immunostaining in IC specimens would allow for using K13 as a diagnostic maker of conjunctival epithelium in immunocytology. The sensitivity of K13 as a marker of non-corneal epithelial cells will need to be determined on pathologic IC specimens.

In addition to the overexpression of *K13* and *K19* in the conjunctiva, two members of the S100 calcium binding protein family, S100A8 and S100A9, were highly expressed in the conjunctiva. S100 proteins function as calcium sensors and upon activation, regulate various cell processes in the epidermis [26]. It has been shown that S100A8 and S100A9 are normally co-expressed together and in response to wound healing, these proteins are secreted by human keratinocytes [27]. Furthermore, localization of both proteins has been observed in both human conjunctival and pterygial epithelia [24,28], and S100A8/9 can also bind to keratin intermediate filaments [29]. Future studies will focus on the specificity of these partner proteins in the conjunctiva.

Another candidate we examined was the carcinoembryonic antigen-related cell adhesion molecule 6 (CEACAM6). CEACAM6 belongs to the carcinoembryonic antigen gene family and has been shown to be localized on epithelia in intestinal cells [30]. Preliminary immunostaining showed localization only in the superficial layer of the conjunctiva and limbus and expression in the cornea was absent (data not shown). Despite being specific for the conjunctiva and limbus, the lack of expression in the basal and suprabasal layers of the epithelia makes this protein an undesirable marker.

Although not listed in Table 3, the mucin family had several genes upregulated in the conjunctiva based on the microarray data, including mucin 5AC and mucin 1. A previous report proposed that mucin 1 (MUC1) could be used as a new marker of conjunctival epithelial cells [13]. However, others have shown that expression of MUC1 is uniform throughout the entire human ocular surface [18,31]. Further study is needed to determine the expression specificity of MUC1 and to resolve this discrepancy before MUC1 could be accepted as a marker of conjunctival epithelial cells.

In summary, the findings of our study show that K13 expression is specific to non-corneal epithelial cells, particularly conjunctival epithelial cells on the healthy ocular surface and K19 is present at substantial levels in the corneal epithelium. The K13 expression pattern and that of K12 are mutually exclusive. This unique feature of K13 makes it a potential candidate as a diagnostic marker to detect the invasion of conjunctival epithelial cells onto the cornea, a hallmark of LSCD.
